# AGEISM IN SUB-SAHARAN AFRICA: PROFESSIONALS’ PERSPECTIVES

**DOI:** 10.1093/geroni/igad104.2320

**Published:** 2023-12-21

**Authors:** Dolapo Adeniji, Abraham Teshome, Gifty Ashirifi, Margaret Adamek

**Affiliations:** Indiana State University, Avon, Indiana, United States; Indiana University, Indianapolis, Indiana, United States; Indiana University, Indianapolis, Indiana, United States; Indiana University, Indianapolis, Indiana, United States

## Abstract

While ageism is increasing across the globe, older adults in Sub-Saharan Africa are more vulnerable to ageism and are often denied a stable means of livelihood leading to poor health and a decreased quality of life. Scholars report that ageism can be experienced in multiple forms including economic, employment, and healthcare, with the intersectionality of gender and other factors complicating ageism. As little is known about ageism in Sub-Saharan Africa, this study explored the perspectives of scholars and professionals about the levels of ageism and age discrimination against older Africans. A cross-sectional study was conducted with scholars and professionals from sub-Saharan Africa. The 78 respondents ranged in age from 24-75 and over half (55.2%) were female. Using descriptive analysis, our findings show that the perceived level of discrimination against older adults is moderate with over half of the respondents (55.7%) indicating that ageism is getting worse. Respondents indicated that older adults are most discriminated against and face barriers in the areas of economic advantages (72.1%), employment opportunities (60.3%), healthcare access (49.4%), and housing (35.3%). Social workers, practitioners, and policymakers should directly address the problem of ageism to promote the healthy living of older adults in sub-Saharan Africa. To turn the tide of ageism and age discrimination in Sub-Saharan Africa, governments need to provide basic social protection to older adults so that they can have a stable means of support in their later years and will be less likely to be perceived as a burden to families and societies.

